# Parametric simulation of micropolar fluid with thermal radiation across a porous stretching surface

**DOI:** 10.1038/s41598-022-06458-3

**Published:** 2022-02-15

**Authors:** Muhammad Bilal, Anwar Saeed, Taza Gul, Wiyada Kumam, Safyan Mukhtar, Poom Kumam

**Affiliations:** 1 Department of Mathematics, City University of Science and Information Technology, Peshawar, 25000 Pakistan; 2 Present Address: Center of Excellence in Theoretical and Computational Science (TaCS-CoE), Faculty of Science, King Mongkut’s University of Technology Thonburi (KMUTT), 126 Pracha Uthit Rd., Bang Mod, Thung Khru, Bangkok, 10140 Thailand; 3 Present Address: Applied Mathematics for Science and Engineering Research Unit (AMSERU), Program in Applied Statistics, Department of Mathematics and Computer Science, Faculty of Science and Technology, Rajamangala University of Technology Thanyaburi (RMUTT), Pathum Thani, 12110 Thailand; 4 Department of Basic Sciences, Deanship of Preparatory Year, King Faisal University, Al Ahsa, Hofuf, Saudi Arabia; 5 Department of Medical Research, China Medical University Hospital, China Medical University, Taichung, 40402 Taiwan

**Keywords:** Engineering, Mathematics and computing

## Abstract

The energy transmission through micropolar fluid have a broad range implementation in the field of electronics, textiles, spacecraft, power generation and nuclear power plants. Thermal radiation's influence on an incompressible thermo-convective flow of micropolar fluid across a permeable extensible sheet with energy and mass transition is reported in the present study. The governing equations consist of Navier–Stokes equation, micro rotation, temperature and concentration equations have been modeled in the form of the system of Partial Differential Equations. The system of basic equations is reduced into a nonlinear system of coupled ODE's by using a similarity framework. The numerical solution of the problem has been obtained via PCM (Parametric Continuation Method). The findings are compared to a MATLAB built-in package called bvp4c to ensure that the scheme is valid. It has been perceived that both the results are in best agreement with each other. The effects of associated parameters on the dimensionless velocity, micro-rotation, energy and mass profiles are discussed and depicted graphically. It has been detected that the permeability parameter gives rise in micro-rotation profile.

## Introduction

The transfer of heat along thin film flow of micropolar fluid has a great impact on research in the field of electronics and especially the exchange of heat inside the circuits of electronic devices, due to uncountable applications described in^1^. To maximize and improve the allowance of heat transfer of patterns flow, extension in the surface flow has been highly effective. Heat transmission is extremely important in industries such as vehicles, textiles, and machines, as well as in the design of all industrial equipment, such as jets, army emanations, spacecraft, turbines of various power generation, and nuclear power plants^2^. To examine the impacts of radiations on the boundary layer of fluids is not an easy job to deal. The phenomenon of heat transfer was explained by Cengel^3^, in the encyclopedia of energy engineering and technology. Khoshvaght et al*.*^4^ explored the dynamics of flow and heat exchange on Sinusoidal-Corrugated tubes computationally. The Micro polar fluid was first introduced by Eringe^5^, who explains the micro-rotation effects on the micro-structures because the theory presented by Navier and stokes does not explains, precisely the properties associated with polymeric fluids, colloidal fluids, suspension and solutions, liquids containing crystals and fluids with additives. Stokes^6^ presented a theoretical approach to fluid flow with micro characteristics. Researchers are studying the effects of radiations on boundary layer of fluids over plates, The thermal radiations effect on micropolar conducting fluid across a uniform expanding surface is reported by Abo-Eldahab et al*.*^7^. Micropolar fluids with heat transition across a permeable medium under the consequences of radiations are discussed by Abo-Eldahab^8^. Ramesh et al*.*^9^ used chemical processes and activation energy effects to transmit the flow, heat, and mass transfer characteristics of a hybrid nanofluid across parallel surfaces. The consequences of viscous resistance on the boundary of the flow, with inertia force and heat transfer in a constant porosity was addressed by Reddy et al*.*^10^. Jyothi et al*.*^11^ and Kumar et al*.*^12^ examined the free convective flow of Maxwell nanofluid across a stretched sheet. The skin friction factor for the Maxwell component is larger than for the Newtonian fluid, and the local Nusselt number is lower for linear radiation and higher for non-linear radiation. Soundalgekar et al*.*^13^ explains the stream and exchange of warm over a ceaselessly moving plate. Gorla et al*.*^14^ investigated the steady heat propagation in micro polar fluid using similarity techniques. The convection micropolar fluid flow and heat propagation characteristics over a vertical surface are studied by Rees et al*.*^15^. Gireesha & Ramesh^16^ used the Runge–Kutta–Fehlberg order approach to analyse the heat of a generalised Burgers nanofluid over a stretched sheet. Ramesh et al*.*^17^ investigated the dusty fluid's 2D boundary layer flow across a stretched sheet. The rate of heat transmission is calculated and presented for a variety of parameter values.

Electromagnetic radiation known as thermal radiation is in the wavelength range of 0.1 to 100 um produced by all matter at a non-zero temperature. It covers a portion of the ultraviolet spectrum as well as all infrared and visible light. At elevated heat (over 1000 K) and following material implosion, when some objects are in clear view of heated debris situated below, radiation heat transmission across parallel sheets becomes significant. Because there aren't any suitable radiative heat exchange models, the energy transition across the plates will be inaccurate^18^. Mahanthesh and Mackolil^19^ investigate the heat propagation of a nanofluid over a plate surface by using quadratic thermal radiation. According to the findings, the density variation with energy differences has huge importance in thermal processes such as solar collectors. Kumar et al.^20^*.* utilised a computational model that included thermal radiation, magnetic field and viscous dissipation to simulate the heat transmission and nanofluid flow along vertical infinite plate. It was realized that improving the value of the radiation constant enriches the energy and velocity profiles. Khader and Sharma^21^ examined the effects of non-uniform heat source and thermal radiation on MHD micropolar fluid flow over a shirking sheet. It has been discovered that the increment in thermal radiation coefficient and micro-polar constant enhances the fluid velocity.

Complex boundary value equations that cannot be resolved are common in the engineering industries. For many systems that are routinely addressed by other computational models, convergence is susceptible to the relaxation constants and starting strategy. The PCM's objective is to investigate the method's universal applicability as a sustainable solution to nonlinear issues^22^. The 3D irregular fluid and heat dispersion over the surface of a rough stretchy spinning disc was highlighted by Shuaib et al*.*^23^. In addition to the influence of external magnetic field, the fluid has been investigated. Shuaib et al*.*^24^ found the property of an ionic transitional boundary layer flow across a revolving disc. Wang et al*.*^25^ Khan reported a parametric continuation algorithm-based stability assessment of nonlinear systems for engineering disciplines. They also investigated the bifurcation that occurs while solving nonlinear IVPs with distinct features and developed an algorithm for determining the bifurcation points in real time.

In our study, we explored the heat exchange in a micropolar fluid with the impacts of radiation across a permeable medium. The problem has been arranged in the form of PDEs (Navier Stokes, energy and concentration equation). The PDEs system has been diminished into the system of ODEs using similarity framework. Which are numerically solved via PCM technique. For this purpose, the modeled equations are tackled numerically by using two different numerical techniques, predictor corrector method and bvp4c method. The obtained conclusions are compared and discussed with the help of graphs, which shows reasonable settlement with each other.

## Mathematical formulation

**Figure 1:**
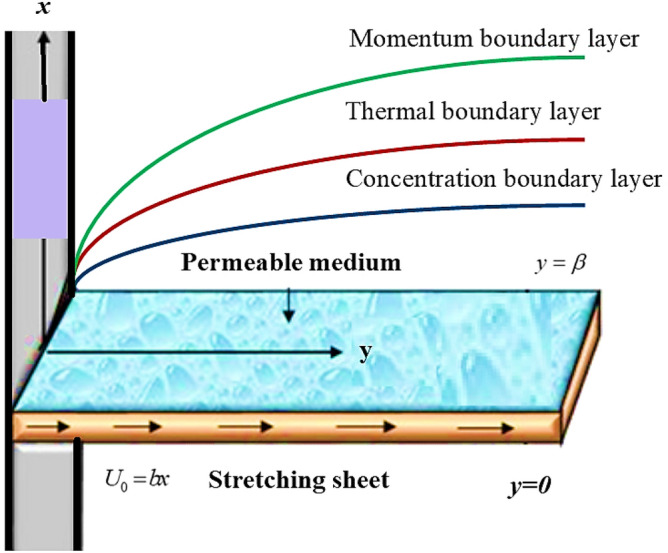
The fluid flow over a stretching surface.

Considered the micropolar fluid flow across a stretched plate with velocity $U_{0} = bx,$. The uniform stretching rate is specified by b > 0, along the *x*-direction. Let d, be the thickness of the surface. The medium is assumed to be permeable over an infinite horizontal sheet in the region $y > 0$ as illustrated in Fig. 1. The thermal radiations effect has been considered along an x-coordinate. Under the above-mentioned presumptions, the flow problem in the of PDEs can be stated as^26,27,29^:

1 $$\frac{\partial u}{{\partial x}} + \frac{\partial v}{{\partial y}} = 0,$$

2 $$u\frac{\partial u}{{\partial x}} + v\frac{\partial u}{{\partial y}} = \nu \frac{{\partial^{2} u}}{{\partial y^{2} }} + k_{c} \frac{\partial \sigma }{{\partial y}} + \frac{\nu \varphi }{K}(U - u) + C_{r} \varphi (U^{2} - u^{2} ),$$

3 $$G_{1} \frac{{\partial^{2} \sigma }}{{\partial^{2} y}} - 2\sigma - \frac{\partial u}{{\partial y}} = 0,$$

4 $$u\frac{\partial \tau }{{\partial x}} + v\frac{\partial \tau }{{\partial y}} = \frac{k}{{\rho c_{p} }}\frac{{\partial^{2} \tau }}{{\partial^{2} y}} + \frac{{16\sigma^{ * } }}{{3\rho c_{p} k^{ * } }}\frac{{\partial^{2} \tau }}{{\partial^{2} y}} + \frac{{vD_{m} k_{T} }}{{T_{m} c_{s} c_{p} }}\frac{{\partial^{2} C}}{{\partial^{2} y}},$$

5 $$u\frac{\partial C}{{\partial x}} + v\frac{\partial C}{{\partial y}} = D_{m} u\frac{{\partial^{2} C}}{{\partial^{2} y}} + \frac{{D_{m} k_{T} }}{{T_{m} }}\frac{{\partial^{2} \tau }}{{\partial^{2} y}}.$$

Boundary conditions for the two-dimensional flow is given as^26,27^:

6 $$\left. \begin{gathered} u = U_{0} ,\,\,\,v = 0,\,\,\,\sigma = 0,\,\,\,\tau = \tau_{w} ,\,\,\,C = C_{w} \,\,\,{\text{at}}\,\,\,y = 0, \hfill \\ \,\,\,\,\,\,\,\,\,\,\,\,\,\,\,\,\,\,\,\,u_{y} = \,\sigma_{y} = \,\,\tau_{y} = C_{y} = 0,\,\,\,v = \delta_{x} \,{\text{at}}\,\,\,y \to 0, \hfill \\ \end{gathered} \right\}$$

Here, the thermal radiation term is defined as:

7 $$q_{r} = \frac{{4\sigma^{ * } }}{{3K^{ * } }}\frac{{\partial \tau^{4} }}{\partial y},$$

While, ignoring the higher order terms in Taylor’s series, we consider only $\tau_{4}$ about $\tau_{1}$, which can be expressed as:

8 $$\tau^{4} \simeq 4\tau_{1}^{3} \tau^{*} - 3\tau_{1}^{4} .$$

by using Eqs. (7) and (8), Eq. (4) becomes,

9 $$u\frac{\partial \tau }{{\partial x}} + v\frac{\partial \tau }{{\partial y}} = \frac{k}{{\rho c_{p} }}\frac{{\partial^{2} \tau }}{{\partial^{2} y}} + \frac{{16\sigma^{ * } \tau_{1}^{3} }}{{3\rho c_{p} k^{ * } }}\frac{{\partial^{2} \tau }}{{\partial^{2} y}} + \frac{{vD_{m} k_{T} }}{{T_{m} c_{s} c_{p} }}\frac{{\partial^{2} C}}{{\partial^{2} y}},$$

The corresponding similarity transformations are^26^:

10 $$\begin{gathered} \psi (x,y) = (2vU_{0} x)^{\frac{1}{2}} f(\eta ),\,\,\,u = \psi_{y} ,\,\,\,v = - \psi_{x} , \hfill \\ \sigma = \left( {\frac{{U_{0} }}{2vx}} \right)^{\frac{1}{2}} U_{0} g(\eta )\,\,{\text{and}}\,\,\eta = \left( {\frac{{U_{0} }}{2vx}} \right)^{\frac{1}{2}} y. \, \hfill \\ \end{gathered}$$

The temperature and concentration for the thin film flow are

11 $$\theta (\eta ) = \left( {\frac{{\tau - \tau_{\infty } }}{{\tau_{w} - \tau_{\infty } }}\,} \right)\,\,\,\,\,\,\,{\text{and}}\,\,\,\,\,\,\,\phi (\eta ) = \left( {\frac{{C - C_{\infty } }}{{C_{w} - C_{\infty } }}} \right)\,.$$

In the consequences of Eq. (10) and Eq. (11) in Eqs. (1)–(6), we get:

12 $$f^{\prime \prime \prime } + \Delta g^{\prime } + ff^{\prime \prime } + \frac{1}{M}(1 - f^{\prime } ) + (1 - f^{\prime 2} ) = 0,$$

13 $$Grg^{\prime \prime } - 2(2g + f^{\prime \prime } ) = 0,$$

14 $$(4 + 3R)\theta^{\prime \prime } + 3R\Pr (Du\phi^{\prime \prime } + f\theta^{\prime } ) = 0,$$

15 $$\phi^{\prime \prime } + Sr\theta^{\prime \prime } - Scf\phi^{\prime } = 0.$$

The system of ODEs transforms boundary conditions are:

16 $$\left. \begin{gathered} f(0) = g(0) = f(0) = \theta (0) = \phi (0) = 1\,\,at\,\,y = 0 \hfill \\ f^{\prime \prime } (1) = f(1) = g^{\prime } (1) = \theta^{\prime } (1) = \phi^{\prime } (0) = 0\,\,at\,\,y = 1. \hfill \\ \end{gathered} \right\}$$

where, $\Delta = {{k_{c} } \mathord{\left/ {\vphantom {{k_{c} } v}} \right. \kern-\nulldelimiterspace} v}$ is the coupling parameter, $Nr = {{2\varphi C_{r} u_{0} } \mathord{\left/ {\vphantom {{2\varphi C_{r} u_{0} } a}} \right. \kern-\nulldelimiterspace} a}$ is the inertia coefficient parameter, $M = {{ka} \mathord{\left/ {\vphantom {{ka} {2\varphi v}}} \right. \kern-\nulldelimiterspace} {2\varphi v}}$ is the permeability parameter, $Gr = {{G_{1} a} \mathord{\left/ {\vphantom {{G_{1} a} v}} \right. \kern-\nulldelimiterspace} v}$ denotes micro rotation parameter, $R = {{4\sigma^{ * } \tau_{1}^{3} } \mathord{\left/ {\vphantom {{4\sigma^{ * } \tau_{1}^{3} } {K^{ * } }}} \right. \kern-\nulldelimiterspace} {K^{ * } }}$ denotes radiations parameter, $\Pr = {{\rho vc_{p} } \mathord{\left/ {\vphantom {{\rho vc_{p} } k}} \right. \kern-\nulldelimiterspace} k}$ denotes Prandtl number, $Sc = {v \mathord{\left/ {\vphantom {v {D_{m} }}} \right. \kern-\nulldelimiterspace} {D_{m} }}$ denotes Schmidt number, $Sr = {{D_{m} K_{T} (T_{w} - T_{0} )} \mathord{\left/ {\vphantom {{D_{m} K_{T} (T_{w} - T_{0} )} v}} \right. \kern-\nulldelimiterspace} v}T_{m} (C_{w} - C_{0} )$ denotes Soret number and $D_{u} = {{D_{m} K_{T} (C_{w} - C_{0} )} \mathord{\left/ {\vphantom {{D_{m} K_{T} (C_{w} - C_{0} )} v}} \right. \kern-\nulldelimiterspace} v}T_{m} (T_{w} - T_{0} )$ denotes Dufour number.

The drag force, Nusselt and Sherwood number, which have several physical and engineering interpretations, are determined as:

17 $$C_{f} = \frac{{\tau_{w}^{s} }}{{\rho u_{w}^{2} }},\,\,\,Nu_{x} = \frac{{xq_{w} }}{{K(\tau_{w} - \tau_{\infty } )}},\,\,\,Sh_{x} = \frac{{xq_{m} }}{{D_{m} (C_{w} - C_{\infty } )}}.$$

where $\tau_{w}^{s}$, $q_{w}^{{}}$ and $q_{m}^{{}}$ are the shear stress, heat and mass fluctuation at the surface, which can be rebound as:

18 $$\tau_{w}^{s} = \mu \left( {\frac{\partial u}{{\partial y}}} \right)^{y = 0} ,\,\,\,q_{w} = - \left( {K\frac{\partial \tau }{{\partial y}}} \right)^{y = 0} ,\,\,\,q_{m} = - \left( {D_{m} \frac{\partial C}{{\partial y}}} \right)^{y = 0} .$$

With $\mu$ being the dynamic viscosity, then from (17) and (18) into (17), we get

19 $$C_{f} {\text{Re}}_{x}^{{\tfrac{1}{2}}} = - f^{\prime \prime } (0),\,\,\,Nu_{x} {\text{Re}}_{x}^{{ - \tfrac{1}{2}}} = - \theta^{\prime } (0),\,\,\,Sh_{x} {\text{Re}}_{x}^{{ - \tfrac{1}{2}}} = - \phi^{\prime } (0).$$

Here ${\text{Re}} = \frac{{u_{0} x}}{v}$ is Reynold number.

## Solution procedures

In this section, the basic methodology and step wise solution of PCM technique have been expressed.

***Step 1***

We presented the following variables to reduced system of BVP to first order:

20 $$\zeta_{1} = f,\,\,\,\zeta_{2} = f^{\prime } ,\,\,\,\zeta_{3} = f^{\prime \prime } ,\,\,\,\zeta_{4} = g,\,\,\,\zeta_{5} = g^{\prime } ,\,\,\,\zeta_{6} = \theta ,\,\,\,\zeta_{7} = \theta^{\prime } ,\,\,\,\zeta_{8} = \phi ,\,\,\,\zeta_{9} = \phi^{\prime } .$$

Making use of Eq. (20) in Eqs. (12–15) and (16), we obtained:

21 $$\zeta_{3}^{\prime } \Delta \zeta_{5} + \zeta_{1} \zeta_{3} + \frac{1}{M}(1 - \zeta_{4} ) + N(1 - \zeta_{2}^{2} ) = 0,$$

22 $$Gr\zeta_{5}^{\prime } 2(2\zeta_{4} + \zeta_{3} ) = 0,$$

23 $$(3R + 4)\zeta_{7}^{\prime } - 3R\Pr (Du\zeta_{9}^{\prime } + \zeta_{7} \zeta_{1} ) = 0,$$

24 $$\zeta_{9}^{\prime } + Sr\zeta_{7}^{\prime } - Sc\zeta_{9} \zeta_{1} = 0.$$

The conditions for first order system of differential equations are:

25 $$\left. \begin{gathered} \zeta_{1} (0) = \zeta_{2} (0) = \zeta_{4} (0) = \zeta_{6} (0) = \zeta_{8} (0) = 1,\,\,\,\,at\,\,\,y = 0, \hfill \\ \zeta_{3} (1) = \zeta_{1} (1) = \zeta_{5} (1) = \zeta_{7} (1) = \zeta_{9} (1) = 0,\,\,\,\,\,\,\,at\,\,\,y = 1. \hfill \\ \end{gathered} \right\}$$

***Step 2*** Introducing the parameter p in Eqs. (21–24):

26 $$\zeta_{3}^{\prime } \Delta \zeta_{5} + \zeta_{1} (\zeta_{3} - 1)p + \frac{1}{M}(1 - \zeta_{4} ) + N(1 - \zeta_{2}^{2} ) = 0,$$

27 $$Gr\zeta_{5}^{\prime } 2(2\zeta_{4} - \zeta_{5} + (\zeta_{5} - 1)p + \zeta_{3} ) = 0,$$

28 $$(3R + 4)\zeta_{7}^{\prime } - 3R\Pr (Du\zeta_{9}^{\prime } + (\zeta_{7} - 1)p\zeta_{1} ) = 0,$$

29 $$\zeta_{9}^{\prime } + Sr\zeta_{7}^{\prime } - Sc\zeta_{1} (\zeta_{9} - 1)p = 0.$$

***Step 3 ***Differentiating Eqs. (21–25) by parameter ’p’

30 $$V^{\prime} = AV + R,$$

where, A and *R* is the coefficient matrix and the remainder.

31 $$\frac{{d\zeta_{i} }}{d\tau }$$

where *i* = 1*,* 2*, ………*11*.*

***Step 4*** Apply superposition principle for each term

32 $$V = aU + W,$$

Solve the following two Cauchy problems for each term

33 $$U = aU,$$

34 $$W = AW + R,$$

introducing Eq. (32) in Eq. (30), we obtained

35 $$(aU + W)^{\prime} = A(aU + W) + R,$$

***Step 5 ***Solving the Cauchy problems

In order to solve the Cauchy issues, a numerical implicit approach is employed, as shown below from Eqs. (33) and (34)

36 $$\frac{{U^{i + 1} - U^{i} }}{\Delta \eta } = AU^{i + 1} ,\,\,or\,\,\,\,(I - \Delta \eta A)U^{i + 1} = U^{i} ,$$

37 $$\frac{{W^{i + 1} - W^{i} }}{\Delta \eta } = AW^{i + 1} ,\,\,or\,\,\,\,(I - \Delta \eta A)W^{i + 1} = W^{i} ,$$

from where we obtain the iterative form of the solution

38 $$U^{i + 1} = (I - \Delta \eta A)^{ - 1} U^{i} ,$$

39 $$W^{i + 1} = (I - \Delta \eta A)^{ - 1} (W^{i} + \Delta \eta R).$$

## Results and discussion

**Figure 2:**
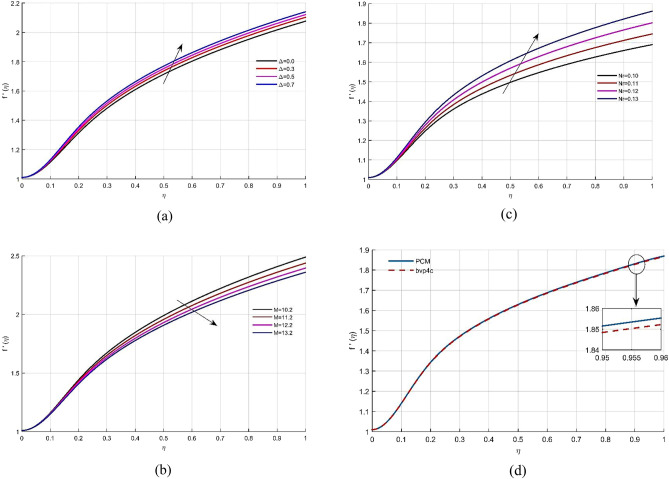
**(a**–**d)** The impact of $\Delta$, *Mr* and *Nr* on non-dimensional velocity field $f^{\prime}(\eta )$. (**d**) Comparison of solution obtained by PCM and bvp4c method.

The thin film flow of micro-polar fluid in permeable media is investigated, as well as the combined influence of temperature and concentration fields across expands in plate. Distinct physical constraints upshot on velocity, energy and concentration profiles have been highlighted. The physical flow behavior is manifested through Fig. 1. Figure 2a evaluates the dependence of the coupling parameter $\Delta$ on the velocity $f(\eta )$. As can be seen, $\Delta$ is inversely linked to the kinematic viscosity of the fluid; as $\Delta$ grows, the thickness drops, and the velocity of the liquid rises.

**Figure 3:**
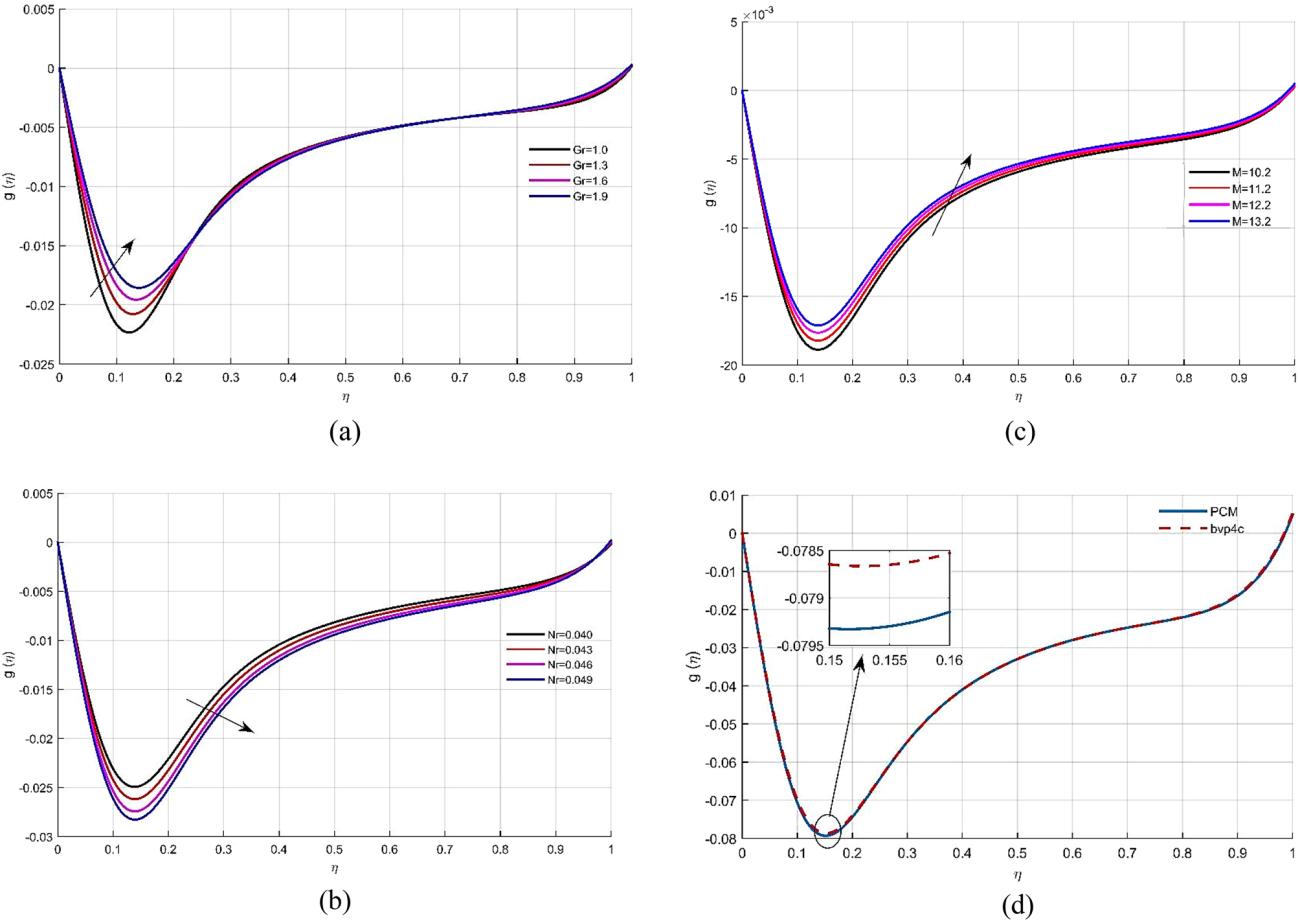
(**a**–**d**) Micro rotation profile $g(\eta )$ under the effects of *Gr*, *Nr* and *Mr*. (**d**) Comparison of solution obtained by PCM and bvp4c method.

Figure 2b depicts the effect of permeability *Mr* on the $f(\eta )$. Given that higher values for *M* resulting in a highly porous media, the fluid flow would obviously decelerate, leading to a drop in velocity. Figure 2c depicts the effect of the inertia coefficient *Nr*. It can be shown that boosting *Nr* credit increases fluid velocity. Figure 2d shows a comparative analysis of the PCM and bvp4c methods vs the velocity field $f(\eta )$.The micro-rotation circular velocity distribution $g(\eta )$ vs different physical constants is represented in Fig. 3a–d. The kinetic energy improves as the value of *Gr* (micro-rotation factor) rises. Physically, when the rotation parameter is elevated, the fluid’s kinematic viscosity drops, and fluid velocity rises. The consequence of the inertia component *Nr* on the radial velocity profile $g(\eta )$ is seen in Fig. 3b. The fluid velocity $g(\eta )$ declines as *Nr* increases. Figure 3c depicts the impact of the permeability element on the non-dimensional micro-rotation angular velocity. Because the permeability factor and the fluid’s viscosity are inversely related, as the permeability parameter increases, the viscosity lowers, and the radial velocity improves. Figure 3d illustrates the comparison of both strategies vs $g(\eta )$.

**Figure 4:**
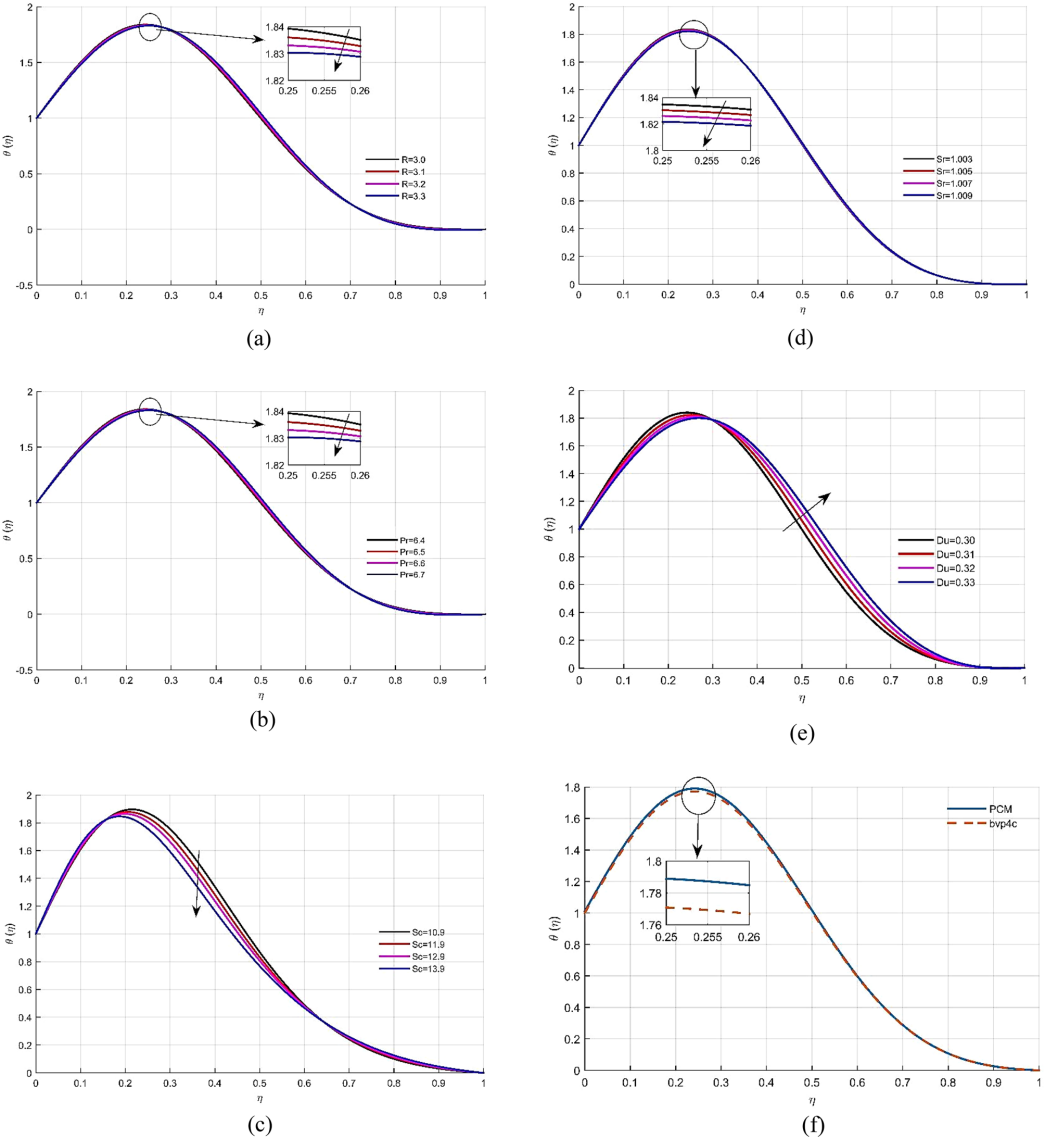
(**a**–**f**) Variation of dimensionless temperature profile $\theta (\eta )$ with parameters *Rd*, *Pr*, *Sc*, *Sr* and *Du* respectively. (**f**) Comparison of solution obtained by PCM and bvp4c method.

The temperature profile $\theta (\eta )$ of the fluid reduces with larger values of the radiation factor *R*, as illustrated in Fig. 4a, The rising effect of radiations reduces the fluid energy profile $\theta (\eta )$. Physically, a fluid with a high Prandtl number has a lower thermal diffusivity. The increase in Pr results in a reduction in $\theta (\eta )$ as displayed in Fig. 4b. Figure 4c shows that increasing the Schmidt number *Sc* lowers the thermal energy $\theta (\eta )$, because Schmidt number effect reduces the boundary layer thickness. The fluid temperature reduces with the action of Soret number *Sr*, as revealed through Fig. 4d. As a result, an enhancement in the Sr corresponds to rises in $\theta (\eta )$. Figure 4e shows the correlation between the Dufour number *Du* and energy profile. The fluid temperature enhances with the positive increment Dufour number *Du*. As demonstrated in Fig. 4f, both solutions for the temperature profile $\theta (\eta )$ have the best correlation.

**Figure 5:**
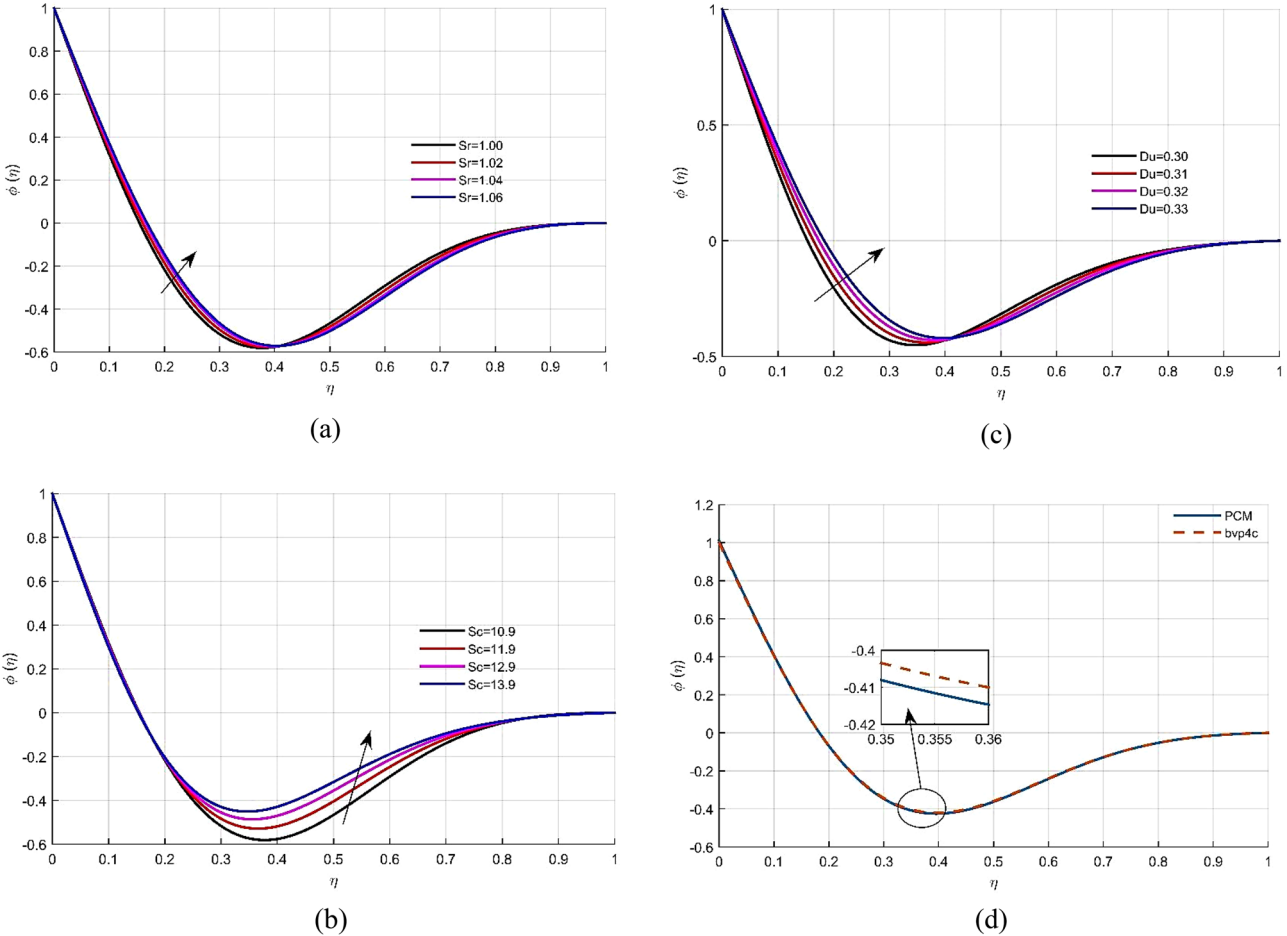
(**a**–**d**) The effects of parameters *Sc*, *Sr* and *Du* on dimensional less concentration profile $\phi (\eta )$ respectively. (**d**) Comparison of solution obtained by PCM and bvp4c method.

Figure 5a explains the response of *Sr* on the concentration allocation $\phi (\eta )$. Because Soret number is directly related to viscosity. The upshot of Schmidt number *Sc* on concentration contour $\phi (\eta )$ is shown in Fig. 5b, which indicates that variation in *Sc* improves the concentration distribution. Figure 5c shows that when the Dufour number *Du* increases, the non-dimensional concentration profile of the liquid grows. As shown in Fig. 5d, the numerical approximation for the concentration gradient $\phi (\eta )$ has the best agreement. Tables 1, 2, 3 provide the numerical results for skin friction, energy transmission, and Sherwood number, as well as a comparison to existing work. Tables 4 and 5 displays the computational estimates for axial velocity, energy, and mass transition profiles for the variation of embedded parameter values.

**Table 1:** Numerical outcomes for skin friction.

$\Delta$	$Mr$	$Nr$	$\left( {C_{f} } \right)$^28^	$\Delta$	$Mr$	$Nr$	$\left( {C_{f} } \right)$
0.2	0.7	0.2	1.37594	0.2	0.7	0.2	1.37589
0.3	0.7	0.2	1.37571	0.3	0.7	0.2	1.37568
0.4	0.7	0.2	1.37547	0.4	0.7	0.2	1.37551
0.2	0.7	0.2	1.37594	0.2	0.7	0.2	1.37587
0.2	0.8	0.2	1.25938	0.2	0.8	0.2	1.25921
0.2	0.9	0.2	1.16533	0.2	0.9	0.2	1.16511
0.2	0.7	0.2	1.37594	0.2	0.7	0.2	1.37575
0.2	0.7	0.3	1.46338	0.2	0.7	0.3	1.46366
0.2	0.7	0.4	1.55067	0.2	0.7	0.4	1.55053

**Table 2:** Numerical outcomes for Nusselt number.

$R$	$Pr$	$Nu_{x}$^28^	$R$	$Pr$	$Nu_{x}$
0.3	0.7	0.245742	0.3	0.7	0.245756
0.3	0.7	0.241842	0.3	0.7	0.241852
0.3	0.7	0.234106	0.3	0.7	0.234108
0.3	0.7	0.245742	0.3	0.7	0.245746
0.3	0.7	0.324886	0.3	0.7	0.324890
0.3	0.7	0.402525	0.3	0.7	0.402528

**Table 3:** Numerical outcomes for Sherwood number.

$\Delta$	$Sc$	$Sh_{x}$^28^	$\Delta$	$Sc$	$Sh_{x}$
0.3	0.3	0.265463	0.3	0.3	0.265471
0.4	0.3	0.264059	0.4	0.3	0.264066
0.5	0.3	0.262655	0.5	0.3	0.262663
0.3	0.3	0.265463	0.3	0.3	0.265471
0.3	0.4	0.266868	0.3	0.4	0.266878
0.3	0.5	0.268272	0.3	0.5	0.268280

**Table 4:** Comparative analysis between bvp4c and PCM techniques for velocity.

$\eta$	${\text{PCM}}\,\,f^{\prime}(\eta )$	${\text{bvp4c}}\,\,f^{\prime}(\eta )$	Absolute error
0.0	$5.09 \times 10^{ - 21}$	0.000000	$5.09 \times 10^{ - 21}$
0.1	0.059923	0.121043	$4.2 \times 10^{ - 7}$
0.2	0.159901	0.211168	$1.7 \times 10^{ - 6}$
0.3	0.259954	0.311364	$3.7 \times 10^{ - 6}$
0.4	0.359994	0.411624	$6.03 \times 10^{ - 6}$
0.5	0.459987	0.511937	$9.2 \times 10^{ - 6}$
0.6	0.559998	0.612295	$1.4 \times 10^{ - 5}$
0.7	0.659935	0.713689	$1.7 \times 10^{ - 5}$
0.8	0.759957	0.813110	$2.3 \times 10^{ - 5}$
0.9	0.859902	0.913549	$2.7 \times 10^{ - 5}$
10.0	0.959946	1.023997	$2.8 \times 10^{ - 5}$

**Table 5:** Comparison between PCM and bvp4c techniques for concentration.

$\eta$	${\text{PCM}}\,\,\phi (\eta )$	${\text{bvp4c}}\,\,\phi (\eta )$	Absolute error
0.0	$1.000000091$	1.000000	$9.1 \times 10^{ - 4}$
0.1	0.8879855	0.903110	$4.2 \times 10^{ - 4}$
0.2	0.709875	0.717920	$7.04 \times 10^{ - 4}$
0.3	0.633047	0.641694	$7.6 \times 10^{ - 4}$
0.4	0.568142	0.575623	$6.4 \times 10^{ - 4}$
0.5	0.514767	0.519828	$4.1 \times 10^{ - 4}$
0.6	0.472423	0.474360	$1.9 \times 10^{ - 4}$
0.7	0.440544	0.439201	$1.3 \times 10^{ - 4}$
0.8	0.418529	0.414291	$3.2 \times 10^{ - 4}$
0.9	0.405765	0.489496	$5.2 \times 10^{ - 4}$
10.0	0.401644	0.483614	$6.03 \times 10^{ - 4}$

## Conclusion

The mass and heat propagation through steady flow of micropolar fluid across a stretched permeable sheet have been analyzed. The modeled equations are numerically computed via PCM technique. The findings are verified with a Matlab source code called bvp4c to ensure that the outputs are accurate. Physical constraints have been explored in relation to velocity, temperature and concentration profiles. The following conclusion may be formed based on the findings of the aforementioned study:

- The PCM and bvp4c approaches are thought to be particularly efficient and reliable
- in determining numerical solutions for a wide range of nonlinear systems of partial differential equations.
- The permeability parameter *M* controls the mobility of the fluid particles, which result in lowering its velocity.
- The thermal radiation and Prandtl number show positive effect on the fluid temperature.
- With increasing credit of Schmidt number *Sc*, the thermal energy profile improves but the mass transmission rate reduces.
- The coefficient of skin friction rises when the radiation parameter and permeability parameter are elevated.

## Abbreviations

*x, y*: Velocity component
$$U_{0} = bx$$: Stretching velocity
$$\Delta$$: Coupling parameter
*Nr*: Inertia coefficient
*M*: Permeability parameter
*Sc*: Schmidt number
*R*: Thermal radiation
*Pr*: Prandtl number
*Sr*: Soret number
*G*: Microrotation parameter
*Du*: Dufour number
*Cf*: Skin friction
*Nu*_*x*_: Nusselt number
*Sh*_*x*_: Sherwood number
*Re*: Reynold number
*PCM*: Parametric continuation method
*BVP*: Boundary value problem
*PDE*: Partial differential equation
*ODE*: Ordinary differential equation
$$f(\eta ),\,g(\eta )$$: Dimensionless velocity
$$\phi (\eta )$$: Dimensionless concentration
$$\theta (\eta )$$: Dimensionless temperature
bvp4c: Matlab built-in package
